# Ecological Role of Volatile Organic Compounds Emitted by *Pantoea agglomerans* as Interspecies and Interkingdom Signals

**DOI:** 10.3390/microorganisms9061186

**Published:** 2021-05-31

**Authors:** Maria Vasseur-Coronado, Anthi Vlassi, Hervé Dupré du Boulois, Rainer Schuhmacher, Alexandra Parich, Ilaria Pertot, Gerardo Puopolo

**Affiliations:** 1 Research and Innovation Centre, Department of Sustainable Agro-Ecosystems and Bioresources, Fondazione Edmund Mach, Via E. Mach 1, 38098 San Michele all’Adige, Italy; mco@scientia.be (M.V.-C.); ilaria.pertot@unitn.it (I.P.); 2 Department of Civil, Environmental and Mechanical Engineering, University of Trento, via Mesiano 77, 38123 Trento, Italy; 3 De Ceuster Meststoffen NV (DCM), Bannerlaan 79, 2280 Grobbendonk, Belgium; hddb@dcm-info.com; 4 Scientia Terrae Research Institute, Fortsesteenweg 30A, 2860 Sint-Katelijne-Waver, Belgium; 5 Department of Agrobiotechnology (IFA-Tulln), Institute of Bioanalytics and Agro-Metabolomics, University of Natural Resources and Life Sciences, Vienna (BOKU), Konrad-Lorenz Straße 20, 3430 Tulln, Austria; anthi.vlassi@boku.ac.at (A.V.); rainer.schuhmacher@boku.ac.at (R.S.); alexandra.parich@boku.ac.at (A.P.); 6 Center Agriculture Food Environment (C3A), University of Trento, via E. Mach 1, 38098 San Michele all’Adige, Italy

**Keywords:** VOC, plant growth-promoting rhizobacteria, *Pantoea agglomerans*, *Pseudomonas putida*, tomato seedlings, dimethyl disulfide

## Abstract

Volatile organic compounds (VOCs) play an essential role in microbe–microbe and plant–microbe interactions. We investigated the interaction between two plant growth-promoting rhizobacteria, and their interaction with tomato plants. VOCs produced by *Pantoea agglomerans* MVC 21 modulates the release of siderophores, the solubilisation of phosphate and potassium by *Pseudomonas* (*Ps.*) *putida* MVC 17. Moreover, VOCs produced by *P. agglomerans* MVC 21 increased lateral root density (LRD), root and shoot dry weight of tomato seedlings. Among the VOCs released by *P. agglomerans* MVC 21, only dimethyl disulfide (DMDS) showed effects similar to *P. agglomerans* MVC 21 VOCs. Because of the effects on plants and bacterial cells, we investigated how *P. agglomerans* MVC 21 VOCs might influence bacteria–plant interaction. Noteworthy, VOCs produced by *P. agglomerans* MVC 21 boosted the ability of *Ps. putida* MVC 17 to increase LRD and root dry weight of tomato seedlings. These results could be explained by the positive effect of DMDS and *P. agglomerans* MVC 21 VOCs on acid 3-indoleacetic production in *Ps. putida* MVC 17. Overall, our results clearly indicated that *P. agglomerans* MVC 21 is able to establish a beneficial interaction with *Ps. putida* MVC 17 and tomato plants through the emission of DMDS.

## 1. Introduction

Plant growth is affected significantly by the intimate interactions established with microorganisms residing in the rhizosphere and, indirectly, on the interactions among these microorganisms. The rhizosphere is indeed a hot spot where microbe–microbe and plant–microbe interactions occur [1,2]. This ecological niche is occupied by a plethora of microorganisms that establish complex interactions with neighbouring microflora, microfauna and plant roots via production of secondary metabolites enabling them to respond and adapt to environmental changes [3].

Ranging from 10^6^ to 10^12^ gene copy numbers/g of sample, bacteria represent the most abundant class of microorganisms residing in soil, plant roots, and rhizosphere [4]. In these regions, bacteria cells regulate their activities through quorum sensing (QS) systems, based on the emission and perception of communication signals [5,6]. These communication signals play an important role in the intraspecies interactions regulating, for example, biofilm formation, biosynthesis of antibiotics, and plasmid conjugal transfer [7,8]. They are also involved in the interspecies interactions by mediating competition and/or cooperativity among bacteria [9,10,11], but they can be sensed also by (micro)organisms belonging to other kingdoms [12]. Among chemical communication signals, N-acyl homoserine lactones (AHLs) received most of the attention so far [13,14]. As AHLs are produced by numerous bacterial species residing in the rhizosphere, they may act as interspecies signals modulating the behaviour of bacterial co-existing populations [9]. At the same time, AHLs can also constitute interkingdom signals and their perception by plants may trigger systemic plant resistance and/or affect plant growth and development [15,16,17].

Volatile organic compounds (VOCs) are a new class of interspecies and interkingdom signal compounds [18]. They are small molecules (<400 Da) belonging to different chemical classes that can evaporate and diffuse easily through air- and water-filled pores [19]. These properties make VOCs ideal signal candidates in the interactions with plant roots. Bacteria were found to produce more than 1000 VOCs and non-organic volatile compounds [20] and some of them increase yield and quality of crop plants. In fact, bacterial VOCs may modulate several physiological processes such as photosynthesis, plant hormone balance [21], uptake of nutrients from soil [22,23], systemic plant resistance mechanisms [24], and tolerance to soil salinization and drought stress [25,26]. At the same time, bacterial VOCs may act as interspecies communication signals playing a relevant role in the cooperation and competition among soilborne bacteria that are not in contact to each other [27]. Indeed, bacterial VOCs may can act as signal molecules between bacteria [28,29] and inhibit or promote the growth of other bacteria [30,31,32]. While the involvement of these chemical compounds in the complex interaction webs of the rhizosphere is largely acknowledged, their specific ecological roles are still far from being fully understood.

As bacterial VOCs are thought to be interspecies and interkingdom communication signals, the aim of our study was to clarify the interaction between two plant growth-promoting rhizobacteria (PGPR) and tomato plants, by analysing the role of the bacterial VOCs in microbe–microbe interaction and, in parallel, their plant growth-promoting effect.

## 2. Materials and Methods

### 2.1. Microorganisms and Plants

The bacterial strains used in this work, *Pantoea agglomerans* MVC 21 and *Pseudomonas* (*Ps.*) *putida* MVC 17, previously isolated from tomato rhizosphere [33], were stored at length in glycerol 40% at −80 °C and routinely grown on Nutrient Agar (NA, Neogen, Miami, FL, USA) in not split Petri dishes (90 mm diameter). To prepare bacterial cell suspensions, bacterial strains were grown in five mL of Nutrient Broth (NB, Oxoid, Basingstoke, UK) at 27 °C on an orbital shaker (200 rpm). After 24 h, a volume of one mL of bacterial cell suspensions was centrifuged (13,000 rpm, 2 min) and pellets were suspended in NaCl solution (0.85% *w/v*) to a final optical density at 600 nm (A_OD600 nm_) of 0.1 corresponding to ≃ 1 × 10^7^ colony forming units (CFU)/mL and used in all the experiments, except when otherwise indicated. 

Tomato seeds (*Solanum lycopersicum* var. Moneymaker; Justseed, Wrexham, UK), surface sterilised according to [33], were sown on not split Petri dishes containing 25 mL of Murashige and Skoog Agar medium (MS, [34]) supplemented with 1.5% (*w/v*) of sucrose (Sigma-Aldrich, St. Louis, MO, USA). Dishes were sealed with double layer of Parafilm tape and kept in a growth chamber (25 ± 1 °C; 70 ± 10% relative humidity (RH); 16 h photoperiod) for 96 h. Four-day-old tomato seedlings were used in all the experiments, except when otherwise indicated.

### 2.2. Evaluation of Compatibility between Pantoea agglomerans MVC 21 and Pseudomonas putida MVC17

A volume of 10 µL of *P. agglomerans* MVC 21 cell suspension was spot-inoculated onto NA in not split Petri dishes. Subsequently, 10 µL of *Ps. putida* MVC 17 cell suspension was spot-inoculated at one cm distance from *P. agglomerans* MVC 21 (Figure S1A). Similarly, the compatibility between the two strains was tested in split Petri dishes (92 mm) with two compartment and ventilation cams (Sarstedt, Nümbrecht, Germany) following the same procedure. The difference consisted in the presence of the separation border of the split Petri dish located in the middle of the 1 cm distance occurring between the two bacterial strains (Figure S1B). Once bacterial strains were spot-inoculated, not split and split Petri dishes were sealed with double layer of Parafilm tape (Beims, Neenah, WI, USA). Monocultures consisting of NA contained in not split and split Petri dishes inoculated solely with *P. agglomerans* MVC 21 or *Ps. putida* MVC 17 were used as untreated controls. After 48 h incubation at 25 °C, dishes were photographed with Bio-Rad Quantity One software implemented in a Bio-Rad Geldoc system (Bio-Rad Laboratories, Inc., Hercules, CA, USA). Digital images were subsequently used to measure the area of bacterial strain macrocolonies using the software ImageJ1.50i [35].

To determine the quantity of *P. agglomerans* MVC 21 and/or *Ps. putida* MVC 17 viable cells residing in the macrocolony, plugs (five mm diameter) were sampled from each macrocolony and transferred into sterile 1.5 mL microcentrifuge tubes containing one mL of sterile NaCl solution (0.85%). After 1 h incubation at 25 °C on an orbital shaker (200 rpm), bacterial cell suspensions were serially diluted (from 10^−1^ to 10^−7^) and those dilutions were plated onto 1/10 Tryptic Soy Agar (TS broth (Oxoid, UK), Agar Technical no.2 16 g/L (Oxoid, UK)). Once inoculated, Petri dishes were maintained at 25 °C and the developed CFUs were counted after 48 h incubation. 

To assess the quantity of cell residing in the macrocolony, the following formula was applied

$$Viable\;cells\;residing\;in\;macrocolony\;(\log_{10})=\;\frac{Counted\;CFU\times Surface\;area\;of\;the\;macrocolony}{Surface\;area\;of\;the\;5\;mm\;plug}$$

Six replicates (Petri dishes) were used for each treatment, and the experiment was repeated.

### 2.3. Assessment of the Interaction Effect on Plant Growth-Promoting Activities of Pantoea agglomerans MVC 21 and Pseudomonas putida MVC 17

The ability of the tested bacterial strains to chelate iron and to solubilise phosphate and potassium was assessed in monoculture and in pairwise combination both on not split and split Petri dishes. Chrome Azurol (CAS) agar, National Botanical Research Institute’s phosphate (NBRIP) medium, and Aleksandrow Agar (AA, HiMedia GmbH, Germany) were used respectively to assess iron chelation and solubilisation of phosphate and potassium according to [33]. In the case of not split Petri dishes, bacterial cell suspensions were spot-inoculated onto AA, CAS agar and NBRIP according to the procedure reported above. In the case of split Petri dishes, one compartment was filled with NA (25 mL) and the second one was filled either with five mL of AA, or CAS agar or NBRIP, respectively. A volume of 10 µL of *P. agglomerans* MVC 21 and/or *Ps. putida* MVC 17 cell suspension was spot-inoculated onto the compartment containing NA. Not split and split Petri dishes were sealed with a double layer of Parafilm tape and incubated at 25 °C. After 48 h, a volume of five µL of *P. agglomerans* MVC 21 and/or *Ps. putida* MVC 17 cell suspension was spot-inoculated onto AA, CAS agar and/or NBRIP contained in the second compartment. Bacterial strains grown as monocultures on AA, CAS agar, and NBRIP were used as untreated controls in the case of not split Petri dishes. Split Petri dishes having the compartment containing NA not inoculated with any bacterial strain were used as untreated controls.

Once inoculated, dishes containing CAS agar were incubated at 25 °C for 24 h whereas dishes containing AA and NBRIP were incubated at 25 °C for 96 h. After each incubation period, not split and split Petri dishes were photographed with Bio-Rad Quantity One software implemented in a Bio-Rad Geldoc system (Bio-Rad Laboratories, USA). In the case of split Petri dishes, ImageJ1.50i was used to measure the area of orange haloes (release of siderophores) and clarification haloes (solubilisation of phosphate and potassium) formed around bacterial macrocolonies. In the case of not split Petri dishes, the haloes were measured on the macrocolony side without contact with the interacting bacterial strain (pairwise combination) or on the corresponding side in the untreated control. For each treatment, six replicates (Petri dishes) were used and the experiment was repeated.

### 2.4. Evaluation of the Effect of VOCs Emitted by Pantoea agglomerans MVC 21 and Pseudomonas putida MVC 17 on Tomato Plant Growth

A volume of 20 µL of *P. agglomerans* MVC 21 and/or *Ps. putida* MVC 17 cell suspension was spot-inoculated into NA contained in one compartment of split Petri dishes. Subsequently, split Petri dishes were sealed with a double layer of Parafilm tape and incubated at 25 °C. After 48 h, three tomato seedlings were placed in the second compartment containing MS medium (25 mL). Tomato seedlings exposed to NA only were used as untreated control. Dishes were sealed with a double layer of Parafilm tape and incubated in a growth chamber (25 ± 1 °C; 70 ± 10% RH; 16 h photoperiod). After 10 days, lateral root density (LRD; number of lateral roots/length main root (cm)) were determined according to [36]. Plant dry weight (mg) was determined after overnight incubation at 65 °C in an incubator. Four replicates (split Petri dishes) were used for each treatment and the experiment was repeated. 

### 2.5. Metabolite Profiling of VOCs Emitted by Pantoea agglomerans MVC 21

#### 2.5.1. Preparation of Samples for Headspace Analysis

For headspace volatile analysis, *P. agglomerans* MVC 21 was inoculated into headspace (HS) vials according to [37] with some modifications. Briefly, five mL of sterilized NA was poured into sterile HS vials (20 mL, La-Pha-Pack, Langerwehe, Germany) and placed horizontally under the laminar flow cabinet. The HS vials were left open overnight at room temperature under the laminar flow cabinet to avoid water condensation. A volume of 20 µL of *P. agglomerans* MVC 21 cell suspension was spot-inoculated onto NA and the HS vials were left to dry under the laminar flow cabinet for at least 2 h. HS vials containing only NA were used as untreated controls in order to distinguish the VOCs emitted by the culture medium. Each HS vial was then tightly sealed with sterile metal caps containing 1.3 mm-silicone/PTFE septa (La-Pha-Pack) and incubated at 25 °C for 144 h before GC-MS measurement. The 144 h time point was chosen as it showed the richest VOC profile of *P. agglomerans* MVC 21 growing in HS vials.

#### 2.5.2. Headspace GC-MS Analysis 

The analysis of VOCs emitted by *P. agglomerans* MVC 21 was performed according to the procedure described by [38]. Briefly, a gas chromatograph (GC) was coupled to a mass selective detector (MSD). The GC-MSD was equipped with a multi-purpose autosampler (MPS), a dynamic headspace system (DHS, Gerstel), a thermal desorption unit (TDU) and a cooled injection system (CIS) unit. Following a 15 min incubation in the DHS at 27 °C, VOCs were dynamically collected from the samples and trapped on a 2 cm TENAX trap at 30 °C. The tenax tube was subsequently dried to remove potentially trapped water. VOCs were then thermally desorbed by heating the TDU unit from 30 °C to 230 °C at a rate of 60 °C/min, followed by a hold time of 5 min and transferred to the GC-MS column by cooled injection in the splitless mode (from −150 °C to 250 °C at a rate of 2 °C/s, hold time 6 min). The chromatographic separation was performed on a HP-MS (5% phenyl methylsiloxane) column (30 m × 0.25 mm × 0.25 µm). The GC oven temperature program was the following: 35 °C for 2 min, raised to 200 °C at 5 °C/ min (hold time 1 min) increased from 200 °C to 250 °C at 20 °C/min (hold time 5 min). Helium was used as carrier gas at a flow rate of one mL/min.

The open-source software MetaboliteDetector [39] version-3.1 (http://metabolitedetector.tu-bs.de/, accessed on 29 May 2021) was used to process the data. The initial parameters used were peak threshold 5.00, peak height 5.00, bins/scan 10 and deconvolution width (scan) 5.00. For compound annotation and identification, a similarity score was calculated with the MetaboliteDetector program by combining the retention index (RI) fit and the similarity of the compound mass spectra. A similarity score ≥0.8 with ΔRI< 5.00 was required. For identification of compounds the spectra and RI values were compared to authentic standards measured under the same GC-MS conditions. Compound annotation was based on comparisons with entries from NIST 14 library (National Institute of Standards and Technology, USA, http://www.nist.gov, accessed on 29 May 2021). The RI was calculated by the software by comparing the experimental retention time to those of a series of n-alkanes (C8-C25) measured under the same chromatographic conditions. Identification levels were assigned according to the criteria described by [40]. 

#### 2.5.3. Preparation of Volatile Organic Compound Solutions for Functional Assays 

Pure 2-phenylethyl alcohol (2PEA), 3-methyl-1-butanol (3M1B) and dimethyl disulfide (DMDS), were purchased (Sigma-Aldrich, USA) and tested for their individual effects on tomato plant growth and on the ability of *Ps. putida* MVC 21 to chelate iron, produce indole-3-acetic Acid (IAA) and solubilise phosphate and potassium.

To determine the dosage to be used for the VOC-mediated functional assays, the amount of each VOC emitted by *P. agglomerans* MVC 21 in the HS vials was estimated as

$$Absolute\;amount\;of\;each\;standard\;(µg)=\;\left(\begin{array}{c} Concentration\;of\;each\;standard\;\left(µg/µL\right)\times \\ \;Volume\;\left(µL\right)of\;standard\;used\;in\;HS\;vial \end{array}\right)\;Calculation\;factor\;of\;each\;standard\;\left(µg/Area\right)=\;\frac{Absolute\;amount\;of\;each\;standard\;\left(µg\right)}{Average\;peak\;area\;measured\;for\;each\;standard}\;Estimated\;amount\;of\;compound\;\left(µg\right)=\;\left(\begin{array}{c} Average\;peak\;area\;measured\;for\;VOC\;in\;the\;GC-MS\;chromatogram\\ \times Calculation\;factor\;of\;each\;compound \end{array}\right)$$

Stock solutions with a concentration of 0.02 ng, 0.2 ng, 2 ng, and 20 ng per split Petri dish of DMDS; 0.09 ng, 0.9 ng, and 9 ng, 90 ng per split Petri dish of 2PEA and 9 ng, 90 ng and 900 ng, 9000 ng per split Petri dish of 3M1B were prepared by serial dilution using ethanol (Merck KGaA, Darmstadt, Germany) to test the effects of pure VOC on plant growth-promoting trait or methanol (Merck, Darmstadt, Germany) to test the effects of pure VOC on tomato plant growth. Ethanol and methanol were used as solvents since they are reported to be nontoxic to bacteria and plants respectively [41,42].

### 2.6. Evaluation of the Effect of Pure VOCs on Tomato Plant Growth and Plant Growth-Promoting Traits of Pseudomonas putida MVC 17

In all experiments, a filter paper (90 mm, VWR) was placed into one compartment of split Petri dishes and inoculated with 20 µL of the stock solutions of 2PEA, 3M1B, and DMDS at different concentrations. In the case of the effect on tomato plant growth, three sterilised and pre-germinated tomato seeds were placed into the second compartment of the split Petri dish containing MS medium following the procedure reported above. To assess the effects of pure VOCs on the ability of *Ps. putida* MVC 17 to release siderophores and solubilise phosphate and potassium, CAS agar, NBRIP or AA medium were poured respectively into the second compartment of the split Petri dish and inoculated with 10 µL of *Ps. putida* MVC 17 as mentioned above. 

*Ps. putida* MVC 17 or pre-germinated tomato seeds exposed to filter papers wetted with ethanol and methanol respectively were used as untreated controls. For each test, split Petri dishes were sealed with a double layer of parafilm tape and incubated following the timing mentioned above. After each respective incubation period, LRD, tomato root, shoot dry weight, and the ability of *Ps. putida* MVC 17 to release siderophores, solubilise of phosphate and potassium were evaluated as mentioned above. To determine whether exposure to pure VOC affected the viability of *Ps. putida* MVC 17 cells, the number of bacterial cells residing in the macrocolony area upon exposure to pure VOC was assessed as mentioned above. Four replicates (split Petri dishes) were used for each treatment and the experiment was repeated. 

### 2.7. Effect of VOCs Released by Pantoea agglomerans MVC 21 on the Interaction between Pseudomonas putida MVC 17 and Tomato Seedlings

A volume of 20 µL of *P. agglomerans* MVC 21 cell suspension was spot-inoculated onto NA (25 mL) contained in one compartment of split Petri dish. Subsequently, dishes were sealed as reported above and incubated at 25 °C. After 48 h, germinated tomato seeds were inoculated with one mL of *Ps. putida* MVC 17 cell suspension according to the procedure described by [33]. Once inoculated, three tomato seeds were placed into the compartment of split Petri dishes containing MS (25 mL). Split Petri dishes having the compartment containing NA not inoculated with *P. agglomerans* MVC 21 and the compartment with MS containing tomato seeds inoculated with *Ps. putida* MVC 17 were used as untreated control. Dishes were sealed with a double layer of Parafilm tape and incubated in the growth chamber as reported above. After 10 days, plant fresh weight, LRD, and plant dry weight were determined. Four replicates (split Petri dishes) were used for each treatment and the experiment was repeated. 

### 2.8. Evaluation of the Effect of Dimethyl Disulfide and Volatile Organic Compounds Emitted by Pantoea agglomerans MVC 21 on the Production of Indole-3-Acetic Acid in Pseudomonas putida MVC 17

IAA production was evaluated according to the procedure described by [33] with modifications. A volume of 20 µL of *P. agglomerans* MVC 21 cell suspension was added into one well of 25-well polystyrene plates (Thermo Fisher Scientific, Waltham, MA, USA) containing three mL of NA. The rest of wells were filled with three mL of DF salt minimal broth amended with 500 µg/mL of L-Tryptophan (Sigma-Aldrich, USA). In the case of DMDS, a sterilised filter paper was placed into one well of 25-well polystyrene plates and was inoculated with 20 µL of a DMDS stock solution (eight mg/mL) to have a final concentration equivalent to 0.02 mg/Petri dish, assuming the complete evaporation of the VOC from the filter paper. Subsequently, the 25-well polystyrene plates were introduced in Petri dishes (150 mm diameter) and sealed with doubled layer of Parafilm tape and kept in the incubator at 25 °C. After 2 days incubation, a volume of 300 µL of *Ps. putida* MVC 17 cell suspension was added to wells containing DF salt minimal broth (three mL) amended with 500 µg/mL of L-Tryptophan. Petri dishes were sealed with a double layer of Parafilm tape and incubated at 28 °C on an orbital shaker (200 rpm). As untreated controls, 25-well polystyrene plates with wells containing non-inoculated NA and/or sterile filter papers were used. After 120 h, the quantity of IAA produced by bacterial cells was monitored according to [33] and expressed as the ratio between A_OD530 nm_ and A_OD600 nm_. For each treatment, six replicates (25-well polystyrene plates) were used and the experiment was repeated.

### 2.9. Statistical Analysis 

All experiments were carried out twice. Normality (Shapiro–Wilk test, *p* > 0.05) and variance homogeneity (Levene’s test, *p* > 0.05) were checked and parametric tests were used. Non-significant differences were found between two experiments (*p* > 0.05) after two-way ANOVA and thus, data from experiments were pooled. Data were subsequently analysed using one-way ANOVA and mean comparisons between treatments were assessed by Tukey’s test (α = 0.05). The statistical analysis was performed using IBM SPSS software (Version 21).

## 3. Results

### 3.1. Compatibility between Pantoea agglomerans MVC 21 and Pseudomonas putida MVC 17

A first set of experiments was carried out to evaluate if *P. agglomerans* MVC 21 and *Ps. putida* MVC 17 might coexist in the same ecological niche. No toxic effect was detected in the compatibility test carried out in not split Petri dishes. Instead, *P. agglomerans* MVC 21 and *Ps. putida* MVC 17 cell density increased significantly when they interacted, compared to when they grew alone (Figure S2A). Similarly, no negative effect of VOCs emitted by *P. agglomerans* MVC 21 and *Ps. putida* MVC 17 was observed on either bacteria, when they interacted in split Petri dishes (Figure S2B).

### 3.2. Interaction with Pantoea agglomerans MVC 21 Modulates the Plant Growth-Promoting Activities of Pseudomonas putida MVC 17

Once assessed the compatibility, experiments were designed to investigate if and how the two bacterial strains might modulate their plant growth-promoting activities during the interaction.

**Figure 1:**
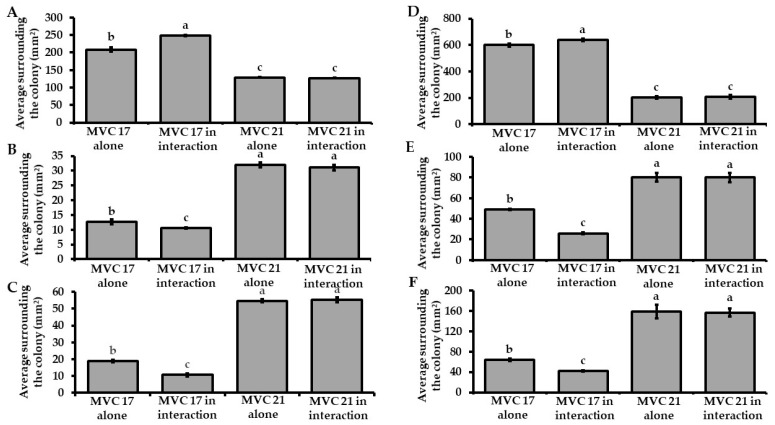
Effect of bacterial interaction on plant growth-promoting activities. Assessment of the ability of *Pantoea agglomerans* MVC 21 and *Pseudomonas* (*Ps*.) *putida* MVC 17 to release siderophores (**A**,**D**) and to solubilise phosphate (**B**,**E**) and potassium (**C**,**F**) during the interaction in not split (**A**–**C**) and split Petri (**D**–**F**) dishes. MVC 17 alone. halo size of *Ps. putida* MVC 17 grown alone; MVC 17 in interaction. Halo size of *Ps. putida* MVC 17 interacting with *P. agglomerans* MVC 21; MVC 21 alone. halo size of *P. agglomerans* MVC 21 grown alone; MVC 21 in interaction. Halo size of *P. agglomerans* MVC 21 interacting with *Ps. putida* MVC 17. Columns represent mean standard error of six replicates (Petri dishes) are reported for each treatment. Data from two independent experiments were pooled. Different letters indicate significant differences among treatments according to Tukey´s test (α = 0.05).

Firstly, no negative effect of VOCs on *P. agglomerans* MVC 21 and *Ps. putida* MVC 17 cell viability was observed in these experiments (Figure S3). In not split Petri dishes, *Ps. putida* MVC 17 significantly increased (19%) the release of siderophores when interacting with *P. agglomerans* MVC 21 compared to the untreated control (Figure 1A). However, the co-inoculum significantly decreased the ability of *Ps. putida* MVC 17 to solubilise phosphate (17%) and potassium (43%) compared to the untreated control (Figure 1B,C). A similar effect was observed with VOCs emitted by *P. agglomerans* MVC 21 that significantly increased the ability of *Ps. putida* MVC 17 to release siderophores (6%) compared to the untreated control (Figure 1D). Conversely, *P. agglomerans* MVC 21 VOCs caused a significant decrease in the ability of *Ps. putida* MVC 17 to solubilise phosphate (47%) and potassium (35%) compared to the untreated control (Figure 1E,F). Differently, *Ps. putida* MVC 17 did not affect the plant growth-promoting activities of *P. agglomerans* MVC 21 compared to the untreated control in both experiments carried out in not split and split Petri dishes (Figure 1A–F). 

### 3.3. Pantoea agglomerans MVC 21 Releases VOCs with a Positive Impact on Tomato Seedling Growth 

The evidence that the VOCs released by *P. agglomerans* MVC 21 modulated the plant growth-promoting activities of *Ps. putida* MVC 17 lead to investigate how and if VOCs released by the two PGPR might affect the growth of tomato seedlings.

**Figure 2:**
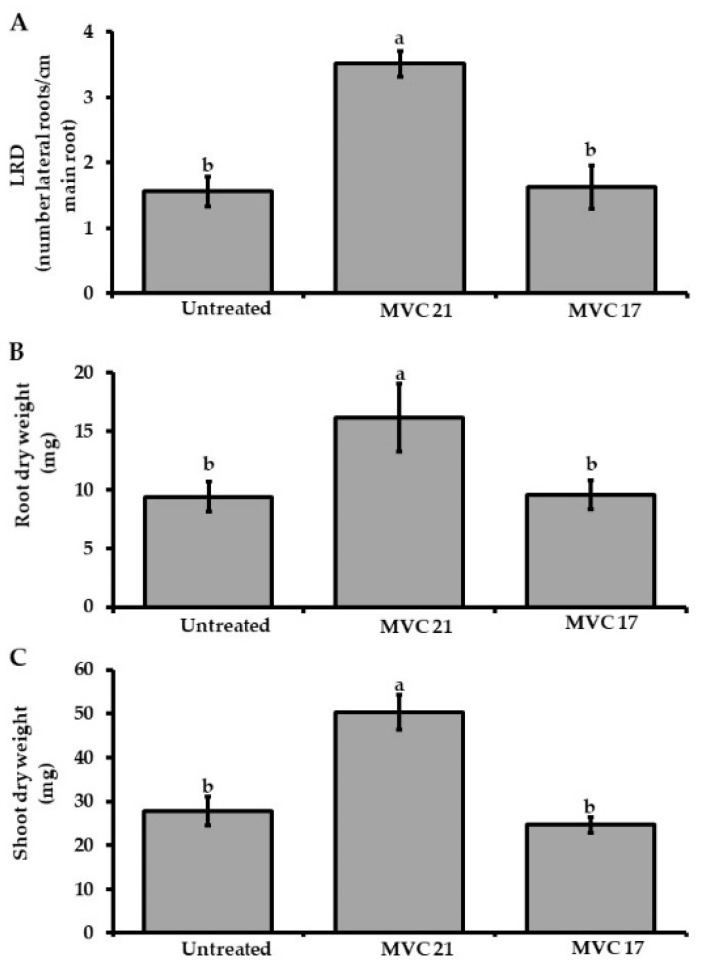
Plant growth-promoting effect of volatile organic compounds (VOCs) emitted by *Pantoea agglomerans* MVC 21 and *Pseudomonas* (*Ps*.) *putida* MVC 17. Lateral Root Density (LRD) (**A**), root (**B**), and shoot (**C**) dry weight of tomato seedlings were assessed after 10 days of exposure to *P. agglomerans* MVC 21 and *Ps. putida* MVC17 VOCs. Untreated. tomato seedlings exposed to agar medium only; MVC 21. tomato seedlings exposed to *P. agglomerans* MVC 21 VOCs; MVC 17. tomato seedlings exposed to *Ps. putida* MVC17 VOCs. Columns represent mean ± standard error values of 12 replicates (tomato seedlings) are reported for each treatment. Data from two independent experiments were pooled. Different letters indicate significant differences among treatments according to Tukey´s test (α = 0.05).

The ability of *P. agglomerans* MVC 21 and *Ps. putida* MVC 17 to stimulate plant growth through the release of VOCs was evaluated in split Petri dishes. Upon exposure to *Ps. putida* MVC 17, no significant changes in LRD, root or shoot biomass were observed (Figure 2A). Conversely, when exposed to *P. agglomerans* MVC 21 VOCs, tomato seedlings significantly increased the LRD (125%), the shoot (71%) and root dry weight (81%) compared to the seedlings exposed to agar medium only (Figure 2B,C).

### 3.4. Headspace Analysis of VOCs Using GC-MS Only Few VOCs Were Produced at Levels Above the Limit of Detection of the Applied GC-MS Method

Based on the positive effect of VOCs released by *P. agglomerans* MVC 21 on tomato seedlings, a headspace analysis was carried out to identify the VOCs having a similar positive effect on tomato seedlings. In total, seven VOCs were identified in the volatile profile of *P. agglomerans* MVC 21 (Table 1). One of them, namely 2-undecanone, was only found in one out of 10 replicate headspace (HS) cultivations. Additionally, some VOCs, namely 2-tridecanone, 2-nonanone, 1-tetradecanol, were found in two and five cultures out of 10, respectively. Three VOCs, namely 2-phenylethyl alcohol (2PEA), 3-methyl-1-butanol (3M1B), and dimethyl disulfide (DMDS), were most consistently (10 out of 10 HS vials) detected. After 96 h of incubation in the HS vial, the estimated amount of each VOC produced by *P. agglomerans* MVC 21 was 2.1 ng of DMDS, 9600 ng of 3M1B and 91.2 ng of 2PEA (Table 1).

### 3.5. Dimethyl Disulfide Shows a Positive Effect on Pseudomonas putida MVC 17 Plant Growth-Promoting Activities and on Tomato Plant Growth

Once identified, the main VOCs produced by *P. agglomerans* MVC 21 were tested to determine the specific effect on *Ps. putida* MVC 17 plant growth-promoting activities and tomato plant growth. Upon exposure to 2PEA, 3M1B, and DMDS, the number of viable cells in *Ps. putida* MVC 17 macrocolony did not change compared to the untreated control (Figure S4). 2PEA and 3M1B did not affect *Ps. putida* MVC 17 plant growth-promoting activities (Tables S1 and S2) whereas DMDS showed modulating activity in a concentration-dependent manner. In particular, DMDS increased the ability of *Ps. putida* MVC 17 to release siderophores at the highest concentration tested (Table 2). In contrast to this, DMDS decreased the ability of *Ps. putida* MVC 17 to solubilise phosphate and potassium (17% and 40%; Table 2).

At the tested concentrations, neither 2PEA nor 3M1B had any effect on tomato seedlings (Tables S1 and S2), whereas DMDS showed a significant increase in the LRD (64%), root (367%), and shoot (239%) dry weight compared to the untreated control (Table 2).

### 3.6. Dimethyl Disulfide and VOCs Emitted by Pantoea agglomerans MVC 21 Affect the Interaction between Pseudomonas putida MVC17 and Tomato Seedlings 

**Figure 3:**
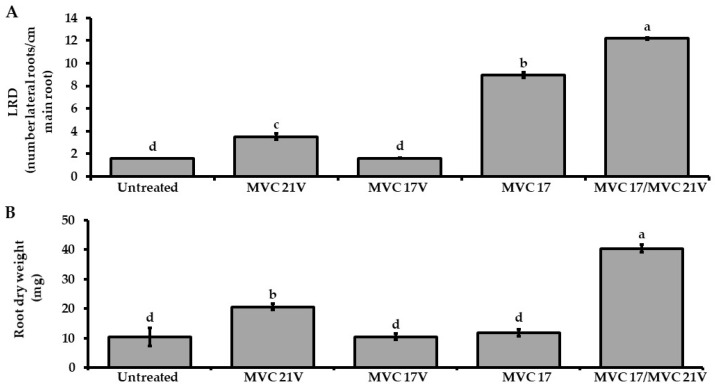
Effect of *Pantoea agglomerans* MVC 21 volatile organic compounds (VOCs) on the plant growth-promoting efficacy of *Pseudomonas (Ps.) putida* MVC 17. Lateral root density (LRD) (**A**) and root dry weight (**B**) of tomato seedlings inoculated with *Ps. putida* MVC 17 were assessed after 10 days of exposure to *P. agglomerans* MVC 21 VOCs. Untreated. tomato seedlings exposed to agar medium only; MVC 21V. tomato seedlings exposed to *P. agglomerans* MVC 21 VOCs; MVC 17V. tomato seedlings exposed to *Ps. putida* MVC 17 VOCs; MVC 17. tomato seedlings inoculated with *Ps. putida* MVC 17; MVC 17/MVC 21V. tomato seedlings inoculated with *Ps. putida* MVC 17 and exposed to *P. agglomerans* MVC 21 VOCs. Columns represent mean ± standard error values of twelve replicates (tomato seedlings) are reported for each treatment. Data from two independent experiments were pooled. Different letters indicate significant differences among treatments according to Tukey´s test (α = 0.05).

Based on the positive effect of the VOCs released by *P. agglomerans* MVC 21 on plant growth of tomato seedlings and plant growth-promoting activities of *Ps. putida* MVC 17, we investigated their effect on the interaction between tomato seedlings and *Ps. putida* MVC 17. Tomato seedlings inoculated with *Ps. putida* MVC 17 significantly increased LRD compared to the untreated control and tomato seedling exposed to *P. agglomerans* MVC 21 VOCs. In contrast, *Ps. putida* MVC 17 did not increase the root dry weight (Figure 3). As reported above, *Ps. putida* MVC 17 had no positive effect on tomato seedling growth, whereas *P. agglomerans* MVC 21 significantly increased tomato LRD and root dry weight compared to the untreated control (Figure 2). Notably, tomato seedlings inoculated with *Ps. putida* MVC 17 and exposed to *P. agglomerans* MVC 21 VOCs significantly increased the LRD and the root dry biomass compared to the untreated control and other treatments (Figure 3A,B).

### 3.7. Dimethyl Disulfide and VOCs Emitted by Pantoea agglomerans MVC 21 Show a Positive Effect on the Production of Indole-3-Acetic Acid by Pseudomonas putida MVC 21 

**Figure 4:**
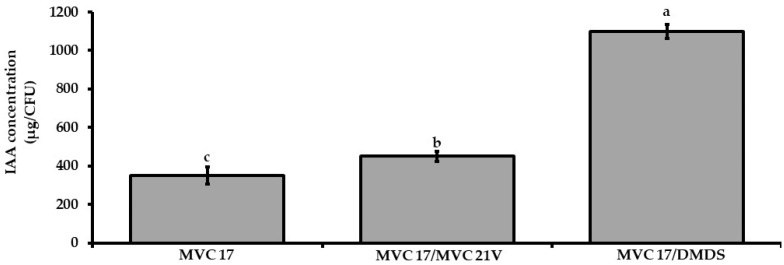
Effect of interaction mediated by volatile organic compounds (VOCs) on indol-3-acetic acid production by *Pseudomonas* (*Ps*.) *putida* MVC 17. The effect of VOCs emitted by *P. agglomerans* MVC 21 and of dimethyl disulfide (DMDS) on the ability of *Ps. putida* MVC 17 to produce indole-3-acetic acid (IAA) was assessed after 120 h of incubation in DF salt amended with L-Tryptophan. MVC 17 alone. IAA production of *Ps. putida* MVC 17 grown alone; MVC 17/MVC 21V. IAA production of *Ps. putida* MVC 17 exposed to *P. agglomerans* MVC 21 VOCs. MVC 17/DMDS. IAA production of *Ps. putida* MVC 17 exposed to DMDS at 0.02 mg/25 well plate. Columns represent mean ± standard error values of six replicates for each treatment. Data from two independent experiments were pooled. Different letters indicate significant differences among treatments according to Tukey´s test (α = 0.05).

As VOCs released by *P. agglomerans* MVC 21 positively influenced the interaction between *Ps. putida* MVC 17 and tomato seedlings, a set of experiments were designed to determine if and how *P. agglomerans* MVC 21 VOCs and DMDS might influence IAA production by *Ps. putida* MVC 17. VOCs emitted by *P. agglomerans* MVC 21 positively affected the production of IAA (29%) by *Ps. putida* MVC 17 compared to the untreated control (Figure 4). Similarly, DMDS positively modulated the production of IAA by *Ps. putida* MVC 17. Particularly, the application of DMDS at 0.02 mg/Petri dish increased the production of IAA by 180% (Figure 4).

## 4. Discussion

Soil bacteria coexist in complex multispecies communities where VOCs play an important role in the interactions among the various microorganisms. In fact, VOCs not only act as antimicrobials by suppressing other microorganisms competing for the same ecological niche, but also as chemical signals, affecting behavior and gene expression of responding microorganisms [43].

Under greenhouse conditions, *P. agglomerans* MVC 21 and *Ps. putida* MVC 17 promote tomato plant growth [33], therefore in this work we aimed at understanding how these PGPR interact among themselves and with tomato seedlings, and how the interaction between the two PGPR may affect the development of tomato seedlings. Firstly, we proved that neither *P. agglomerans* MVC 21 nor *Ps. putida* MVC 17 released any diffusible toxic metabolite or VOC against the other. In fact, the number of *P. agglomerans* MVC 21 and *Ps. putida* MVC 17 viable cells present in interacting macrocolonies grown in not split Petri dishes were even higher compared to monocultures, whereas cell numbers were not significantly differing in the case of split Petri dishes. These results suggest that these two PGPR might coexist under natural conditions and share the same ecological niches when applied in the field. From a practical point of view, this information indicates that these two strains may be combined in a biostimulant product, as already reported for a combination of other PGPR belonging to *P. agglomerans* and *Ps. putida* [44]. From an ecological point of view, our results confirm previous evidences of how members of the genus *Pantoea* and *Pseudomonas* may coexist and interact in the phytobiome. In fact, it was already shown that *P. agglomerans* strains were associated with *Ps. savastanoi* pv. *savastanoi*, the causal agent of olive (*Olea europaea* L.) knot disease, and their interaction determined the increase of olive knot size [45].

These bacterial species were found to interact through a QS mechanism based on the production of AHLs [46]. In our case, interaction experiments carried out in not split and split Petri dishes indicated that the interaction between *P. agglomerans* MVC 21 and *Ps. putida* MVC 17 mainly relied on the ability of *P. agglomerans* to release VOCs. Particularly, the VOCs emitted by *P. agglomerans* MVC 21 were able to affect the ability of *Ps. putida* MVC 17 to release siderophores and solubilise phosphate and potassium. VOCs may affect different bacterial traits as cell motility, biofilm formation [47] cell metabolism, cell wall biosynthesis and response to stresses [48]. At the best of our knowledge, this is the first time that VOCs, as a communication signalling system, were shown to modulate plant growth-promoting activities in PGPR.

Notably, other communication signalling systems are controlling plant growth-promoting activities in bacteria. For instance, the AHL system in *Paracoccus denitrificans* [49], Autoinducer-2 (AI-2) system in *Actinobacillus actinomycetemcomitans* [50] and *Porphyromonas gingivalis* [51], and the *Pseudomonas* quinolone signal (PQS) system in *Ps. aeruginosa* [52] were found to mediate the ability of these bacteria to chelate iron from the environment. Similarly, AHL and AI-2 systems were shown to be involved in phosphorous acquisition in the cyanobacterium *Trichodesmium consortia* [53]. Given the importance of iron and phosphorous in the physiology of bacterial cells, it is conceivable that different communication signalling systems evolved in bacteria to regulate the uptake of these elements from the environment.

Interestingly, we found that the VOCs emitted by *P. agglomerans* MVC 21 were also involved in the establishment of a positive interaction with tomato plants. Indeed, tomato seedlings exposed to *P. agglomerans* MVC 21 VOCs showed a significant increase in LRD, root and shoot dry weight compared to the untreated control and the ones exposed to *Ps. putida* MVC 17 VOCs. The ability of *P. agglomerans* strains to stimulate plant growth through production of siderophores, phytohormones, and solubilisation of phosphate is widely accepted [54,55,56,57,58]. In contrast, investigation on the involvement of VOCs produced by *P. agglomerans* in plant growth promotion is still in its infancy. However, the genome sequencing of plant beneficial *P. agglomerans* strains highlighted the presence of genes being putatively involved in the biosynthesis of VOCs that may stimulate plant growth [54,59]. Moreover, [27] found that the endophyte *P. agglomerans* E44 was able to stimulate seed germination and increase primary root length as well as fresh biomass of wild cabbage seedlings.

VOCs released by *P. agglomerans* E44 belong to the family of alkenes (1-undecene, cyclohexane), sulfides (DMDS and dimethyl trisulfide), and terpenes such as alpha-pinene [27]. In our case, *P. agglomerans* MVC 21 released VOCs belonging to the family of alcohols, ketones and sulfide. More specifically, 2PEA, 3M1B, and DMDS were the VOCs detected in the headspace of all of the tested culture replicates of *P. agglomerans* MVC 21. Furthermore, bioassays testing the effect of pure VOCs indicates that DMDS is the main active VOC released by *P. agglomerans* MVC 21, able to modulate the plant growth-promoting activities of *Ps. putida* MVC 17 and to stimulate the growth of tomato seedlings in a dose-dependent manner. 

Volatile sulfide compounds, such as DMDS, dimethyl sulfide and dimethyl trisulfide play an important role in plant–microbe and interspecific microbe–microbe interactions [30,60]. DMDS is produced by several bacteria as *Bacillus* sp., *Pantoea* sp., *Pseudomonas* sp., *Serratia* sp., and *Stenotrophomonas* sp. [42,43,61]. It is also the main volatile produced by *Serratia plymuthica* IC1270 that suppressed the growth of *Agrobacterium* strains [62]. However, little is known on the impact of DMDS on the production of siderophores and solubilisation of phosphate and potassium by PGPR. Our results showed that DMDS, similarly to *P. agglomerans* MVC 21 VOCs, significantly affected plant growth-promoting activities in *Ps. putida* MVC 17. As DMDS did not show any effect on the viability of *Ps. putida* MVC 17 cells, it is probable that this VOC may modulate expression of genes associated to plant growth-promoting activities, acting as an interspecies signal that allows bacteria to coordinate their behaviour according to neighbouring communities. In the future, transcriptome analysis of rhizosphere associated bacteria might be carried out to understand how DMDS affects their behaviour.

DMDS was the main active compound released by *P. agglomerans* MVC 21 responsible for the promotion of tomato seedlings in a concentration-dependent manner. Interestingly, after exposure to VOCs emitted by *P. agglomerans* MVC 21 and/or pure DMDS, tomato seedlings increased their LRD, as well as root and shoot biomass. DMDS may inhibit plant growth when applied at high concentrations; whereas it may inhibit plant pathogens and induce plant systemic resistance when used at low concentrations [63,64,65]. DMDS produced by *Burkholderia ambifaria* increased *Arabidopsis thaliana* biomass, resistance to gentamicin and kanamycin in *Escherichia coli* and inhibited *Alternaria alternata* and *Rhizoctonia solani* growth [66]. Similar to other VOCs, DMDS may modulate root system architecture by affecting plant hormone biosynthesis. Indeed, indole and DMDS promoted root development by modulating auxin signalling pathways in *A. thaliana*. In particular, DMDS strongly enhanced auxin signalling near the root apical meristem [67,68]. Thus, we can hypothesize that *P. agglomerans* MVC 21 VOCs and DMDS had a similar effect by promoting auxin biosynthesis in tomato seedlings in our system.

As both DMDS and VOCs released by *P. agglomerans* MVC 21 influenced the activities of *Ps. putida* MVC 17 and the growth of tomato seedlings, we hypothesized that they might also influence the interaction between the plant and bacterium. Strikingly, our results showed that VOCs released by *P. agglomerans* MVC 21 positively influenced this interaction by boosting the plant growth-promoting efficacy of *Ps. putida* MVC 17. Indeed, tomato seedlings interacting with *Ps. putida* MVC 17 and exposed to VOCs released by *P. agglomerans* MVC 21 showed a significant increase of LRD and root dry weight. These results lead us to hypothesize that, similarly to what is reported in plants [68], DMDS and *P. agglomerans* VOCs might also have a promoting effect on indole-3-acetic acid production in bacteria. Our results clearly showed that DMDS and *P.* *agglomerans* VOCs enhanced indole-3-acetic acid production in *Ps. putida* MVC 17, providing thus the first evidence of DMDS to be a VOC signal that is able to affect the same biosynthetic pathway in two kingdoms. 

In conclusion, this work suggests that VOCs and, in particular, DMDS emitted by *P. agglomerans* species have an ecological role in the rhizosphere. In fact, VOCs released by *P. agglomerans* may act as chemical signals by modulating the behaviour of tomato plants as well as PGPR belonging to the species *Ps. putida,* even when far from each other. In future, mutants of *P. agglomerans* MVC 21 impaired in VOC production and/or unable to release DMDS might be created to better determine the role played by these compounds in the interaction between *P. agglomerans*, PGPR and crop plants. From a practical point of view, our results show that DMDS, used as a commercial soil fumigant for the control of plant pathogens [18], may stimulate also plant growth and improve the interaction between plants and PGPR residing in agricultural soils.

## Figures and Tables

**Table 1:** Volatile organic compounds (VOCs) detected by gas chromatography-mass spectrometry (GC-MS) in the headspace of vials inoculated with *Pantoea agglomerans* MVC 21 and incubated for six days.

Metabolite ^1^	RI ^2^	Sim Score ^3^	Level of Identification ^4^	HS Vials ^5^
Dimethyl disulfide	735	0.98	1	10/10
3-Methyl-1-butanol	752	0.99	1	10/10
2-Phenylethyl-alcohol	1115	0.97	1	10/10
2-Tridecanone	1498	0.89	1	5/10
2-Nonanone	1093	0.87	1	2/10
1-Tetradecanol	1671	0.91	1	2/10
2-Undecanone	1295	0.87	1	1/10

^1^ VOCs not related to the untreated control. ^2^ Average retention index (RI). ^3^ Average similarity score ≥0.80. ^4^ Levels of identification were assigned according to Blaženović et al. (2018) where 1 = confident identification by comparison of GC-MS spectrum and RI to standard compounds analysed under the same chromatographic and MS conditions. ^5^ Number of sample replicates in which the VOC was detected to the total number of replicates analysed.

**Table 2:** Effect of pure dimethyl disulfide (DMDS) on the plant growth-promoting activities of *Pseudomonas* (*Ps.*) *putida* MVC 17 and tomato plant growth.

DMDS Concentration *	Siderophore Release (mm^2^) **	Phosphate Solubilisation (mm^2^)	Potassium Solubilisation (mm^2^)
0	592.10 ± 2.69 c	27.35 ± 0.79 a	76.37 ± 0.25 a
0.02	610.16 ± 1.25 c	24.77 ± 0.56 a	74.34 ± 0.56 a
0.2	612.33 ± 2.33 c	20.39 ± 0.36 b	66.18 ± 0.36 b
2	675.53 ± 1.09 b	17.21 ± 0.44 b	65.81 ± 0.44 b
20	707.01 ± 2.45 a	16.37 ± 0.74 b	63.30 ± 0.74 b
DMDS Concentration *	Lateral Root Density ***	**Rood Dry Weight (mg)**	**Shoot Dry Weight (mg)**
0	1.46 ± 0.05 c	12.32 ± 0.55 d	31.14 ± 0.92 d
0.02	1.82 ± 0.03 b	26.43 ± 1.33 c	53.31 ± 2.33 c
0.2	1.94 ± 0.06 b	37.87 ± 0.21 c	79.25 ± 1.25 c
2	2.31 ± 0.12 a	47.31 ± 1.23 b	84.25 ± 0.99 b
20	2.40 ± 0.16 a	57.65 ± 2.36 a	102.33 ± 1.22 a

The ability of *Ps. putida* MVC 17 to release siderophores, solubilise phosphate and potassium was assessed after exposure to different concentrations of DMDS. Lateral root density, root, and shoot dry weight were evaluated after 10 days of exposure to different concentrations of pure DMDS. Mean ± standard error values of six replicates are reported for each treatment in the case of plant growth-promoting activities of *Ps. putida* MVC17 whereas mean ± standard error values of 12 replicates (tomato seedlings) are reported for each treatment in the case of tomato plant growth. In both cases, data from two independent experiments were pooled. Different letters indicate significant differences among treatments according to Tukey´s test (α = 0.05). * ng/split Petri dish; ** average halo area surrounding the colony; *** number lateral roots/cm main root.

## Supplementary Materials

The following are available online at https://www.mdpi.com/article/10.3390/microorganisms9061186/s1, Figure S1: Experimental design used to investigate on the interaction between Pantoea agglomerans MVC 21 and Pseudomonas (Ps.) putida MVC 17; Figure S2: Compatibility between Pantoea agglomerans MVC 21 and Pseudomonas (Ps.) putida MVC 17; Figure S3: Viability of Pseudomonas (Ps.) putida MVC 17 cells after exposure to Pantoea agglomerans MVC 21 volatile organic compounds (VOCs); Figure S4: Effect of pure 3-metyl-1-butanol (3M1B), 2-phenylethyl alcohol (2PEA) and dimethyl disulfide (DMDS) on the viability of *Pseudomonas* (*Ps*.) *putida* MVC 17 cells.
