# Supplementary material for: Ecological Role of Volatile Organic Compounds Emitted by Pantoea agglomerans as Interspecies and Interkingdom Signals
Source: Microorganisms. 2021 May 31;9(6):1186. doi: 10.3390/microorganisms9061186 (PMC8229667; doi:10.3390/microorganisms9061186)
Supplement: Supplementary file 1 [file microorganisms-09-01186-s001.zip › Supplementary figure legends.pdf]

Figure S1: Experimental design used to investigate on the interaction between *Pantoea agglomerans* MVC 21 and *Pseudomonas putida* MVC 17. (A) in not split Petri dishes, interactions between *P. agglomerans* MVC 21 and *Ps. putida* MVC 17 were created by spot-inoculating a volume of 10 μl of bacterial cell suspension at 1 cm distance. (B) in split Petri dishes, interactions interactions between *P. agglomerans* MVC 21 and *Ps. putida* MVC 17 were created by spot-inoculating 10 μl of bacterial cell suspension at 0.5 cm distance from the separation border of the split Petri. Growth media included in the not split and split Petri dishes changed according to the specific experiment.

Figure S1: Compatibility between *Pantoea agglomerans* MVC 21 and *Pseudomonas* (*Ps.*) *putida* MVC 17. The quantity of viable cells of *P. agglomerans* MVC 21 and *P. putida* MVC 17 residing in the macrocolonies was assessed after 48 h incubation at 25°C when they grew in common Petri dishes (A) and in split Petri dishes (B). MVC 17 alone. number of *Ps. putida* MVC 17 cells grown alone; MVC in interaction. number of *Ps. putida* MVC 17 cells grown in compatibility with *P. agglomerans* MVC 21; MVC 21 alone. number of *P. agglomerans* MVC 21 cells grown alone; MVC in interaction. number of *P. agglomerans* MVC 21 cells grown in compatibility with *Ps. putida* MVC 17. Columns are mean ± standard error values with six replicates (Petri dishes) are reported for each treatment. Data originating from two independent experiments were pooled. Asterisks indicate significant differences between treatments according to Student´s t-Test ($\alpha = 0.05$).

Figure S2. Viability of *Pseudomonas* (*Ps*). *putida* MVC 17 after exposure to *Pantoea agglomerans* MVC 21 Volatile Organic Compounds (VOCs). The number of *Ps. putida* MVC 17 viable cells grown on different media to detect release of siderophores (A), solubilisation of phosphate (B) and potassium (C) was evaluated upon exposure to *P. agglomerans* MVC 21 VOCs. MVC 17 alone. number of *Ps. putida* MVC 17 viable cells grown alone; MVC 17 in interaction. number of *Ps. putida* MVC 17 viable cells after exposure to *P. agglomerans* MVC 21 VOCs. Columns are mean ± standard error values for six replicates (Petri dishes) were pooled from two independent experiments are reported for each treatment. Asterisks indicate significant differences between treatments according to Student´s t-Test ($\alpha = 0.05$).

Figure S3. Effect of pure 3-metyl-1-butanol (3M1B), 2-phenylethyl alcohol (2PEA) and dimethyl disulfide (DMDS) on the viability of *Pseudomonas* (Ps.) p*utida* MVC 17. The number of *Ps. putida* MVC 17 viable cells grown on media to detect release of siderophores (A-C), solubilise phosphate (D-F) and potassium (G-I) was assessed after exposure to different concentrations of 3M1B (A,D,G), 2PEA (B,E,H) and DMDS (C,F,I) . Columns indicate mean ± standard error values for six replicates (Petri dishes) are reported for each treatment. Data originating from two independent experiments were pooled. Different letters indicate significant differences among treatments according to Tukey´s test ($\alpha = 0.05$).
