# Supplementary material for: Ecological Role of Volatile Organic Compounds Emitted by Pantoea agglomerans as Interspecies and Interkingdom Signals
Source: Microorganisms. 2021 May 31;9(6):1186. doi: 10.3390/microorganisms9061186 (PMC8229667; doi:10.3390/microorganisms9061186)
Supplement: Supplementary file 1 [file microorganisms-09-01186-s001.zip › Supplementary tables.pdf]

Table S1. Effect of pure 3-methyl-1-butanol (3M1B) on the plant growth-promoting activities of *Pseudomonas* (*Ps.*) *putida* MVC 17 and tomato plant growth.

3M1B concentration*	Siderophore release (mm^2)**	Phosphate solubilization (mm^2)	Potassium solubilization (mm^2)	Lateral root density ***	Root dry weight (mg)	Shoot dry weight (mg)
0	557.26 ± 1.45 a	28.79 ± 0.56 a	70.98 ± 1.45 a	1.49 ± 0.20 a	17.65 ± 1.26 a	40.99 ± 1.25 a
9	560.33 ± 2.13 a	29.30 ± 0.15 a	71.79 ± 1.33 a	1.52 ± 0.12 a	18.15 ± 1.23 a	39.13 ± 1.26 a
90	559.12 ± 3.02 a	28.95 ± 0.32 a	71.65 ± 2.25 a	1.59 ± 0.19 a	18.03 ± 2.26 a	40.45 ± 1.09 a
900	563.22 ± 1.26 a	28.77 ± 0.55 a	72.70 ± 1.26 a	1.58 ± 0.14 a	17.99 ± 1.09 a	41.45 ± 0.79 a
9000	562.36 ± 1.33 a	29.23 ± 0.12 a	72.15 ± 1.45 a	1.57 ± 0.19 a	18.63 ± 0.99 a	41.99 ± 1.33 a

The ability of *Ps. putida* MVC 17 to release siderophores, solubilise phosphate and potassium was assessed after exposure to different concentrations of 3M1B. Lateral root density, root and shoot dry weight were evaluated after 10 days of exposure to different concentrations of pure 3M1B. Mean ± standard error values of six replicates are reported for each treatment in the case of plant growth-promoting activities of *Ps. putida* MVC17 whereas mean ± standard error values of twelve replicates (tomato seedlings) are reported for each treatment in the case of tomato plant growth. In both cases, data from two independent experiments were pooled. Different letters indicate significant differences among treatments according to Tukey´s test ($\alpha = 0.05$).

*ng/split Petri dish

**average halo area surrounding the colony

***number lateral roots/cm main root

Table S2. Effect of pure 2-phenylethyl alcohol (2PEA) on the plant growth-promoting activities of *Pseudomonas* (*Ps.*) *putida* MVC 17 and tomato plant growth.

2PEA concentration*	Siderophore release (mm^2)**	Phosphate solubilization (mm^2)	Potassium solubilization (mm^2)	Lateral root density ***	Root dry weight (mg)	Shoot dry weight (mg)
0	558.02 ± 2.56 a	27.99 ± 0.89 a	74.02 ± 2.02 a	1.47 ± 1.01 a	18.99 ± 1.03 a	34.01 ± 1.39 a
0.09	563.23 ± 2.31 a	28.10 ± 1.21 a	74.50 ± 1.23 a	1.45 ± 0.02 a	20.20 ± 1.26 a	33.98 ± 1.26 a
0.9	569.33 ± 1.26 a	28.65 ± 0.98 a	74.74 ± 0.69 a	1.49 ± 0.06 a	20.79 ± 1.03 a	34.02 ± 1.01 a
9	559.12 ± 1.75 a	28.90 ± 2.31 a	74.37 ± 1.06 a	1.50 ± 0.09 a	19.99 ± 1.49 a	33.99 ± 0.77 a
90	561.03 ± 2.02 a	28.75 ± 0.97 a	73.89 ± 1.31 a	1.49 ± 0.12 a	18.52 ± 1.33 a	33.08 ± 0.89 a

The ability of *Ps. putida* MVC 17 to release siderophores, solubilise phosphate and potassium was assessed after exposure to different concentrations of 2PEA. Lateral root density, root and shoot dry weight were evaluated after 10 days of exposure to different concentrations of pure 2PEA. Mean ± standard error values of six replicates are reported for each treatment in the case of plant growth-promoting activities of *Ps. putida* MVC17 whereas mean ± standard error values of twelve replicates (tomato seedlings) are reported for each treatment in the case of tomato plant growth. In both cases, data from two independent experiments were pooled. Different letters indicate significant differences among treatments according to Tukey´s test ($\alpha = 0.05$).

*ng/split Petri dish

**average halo area surrounding the colony

***number lateral roots/cm main root
