# Supplementary figures and images for: Ecological Role of Volatile Organic Compounds Emitted by Pantoea agglomerans as Interspecies and Interkingdom Signals
Source: Microorganisms. 2021 May 31;9(6):1186. doi: 10.3390/microorganisms9061186 (PMC8229667; doi:10.3390/microorganisms9061186)

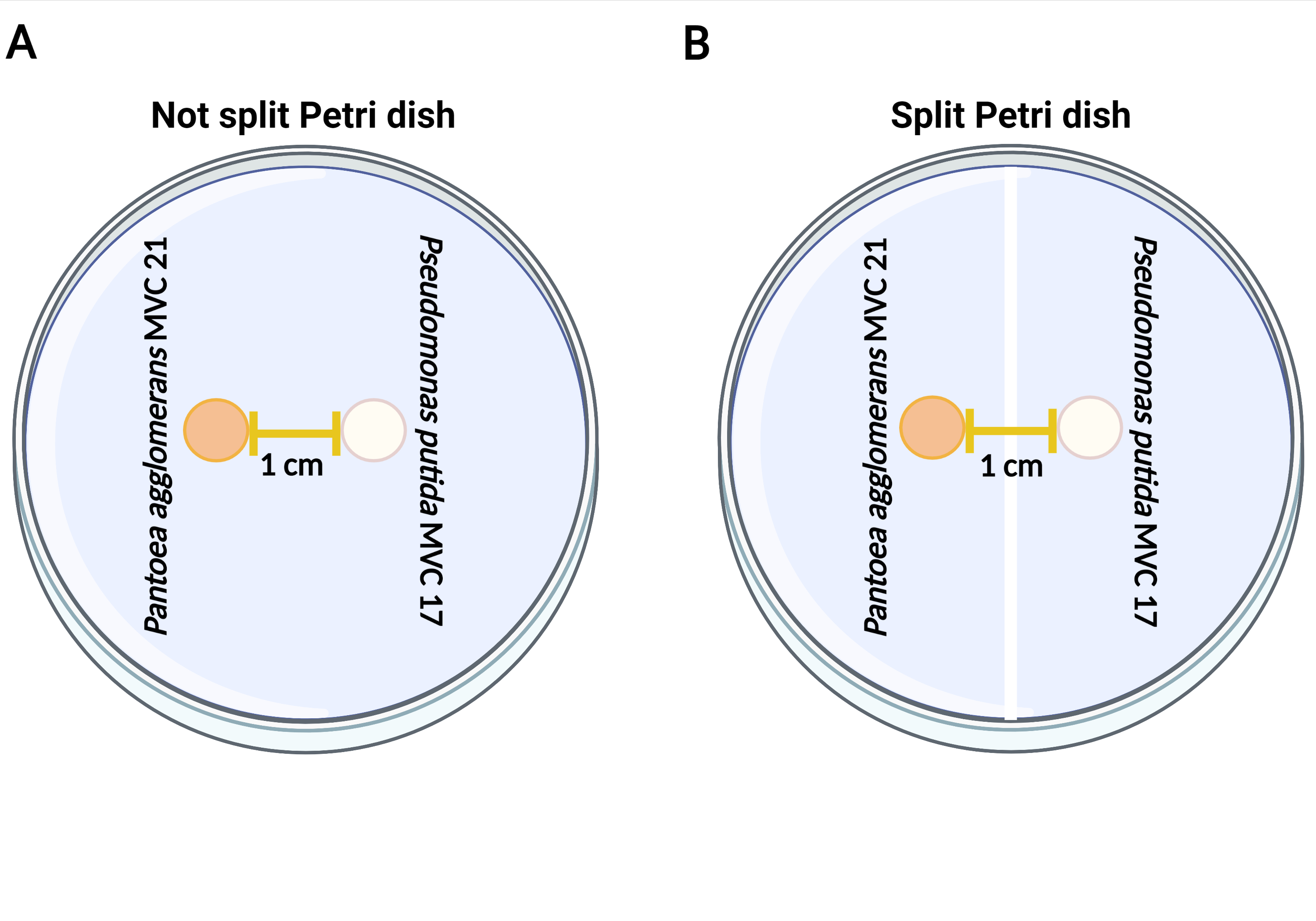

Supplement: Supplementary file 1 [file microorganisms-09-01186-s001.zip › Figure S1.jpeg]

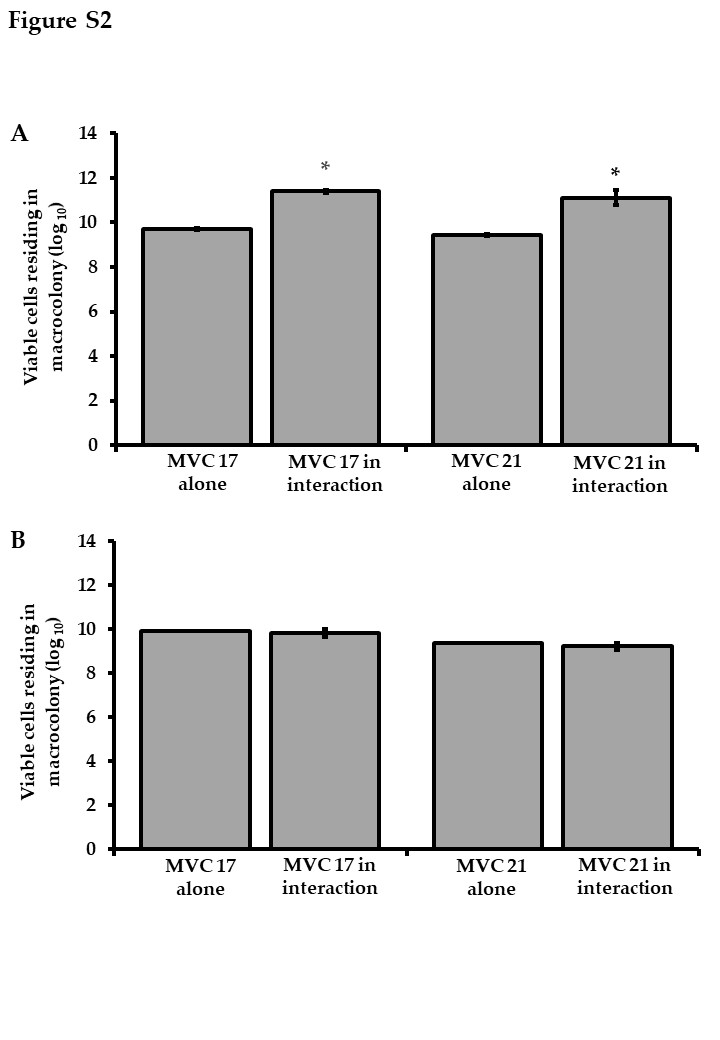

Supplement: Supplementary file 1 [file microorganisms-09-01186-s001.zip › Figure S2.jpg]

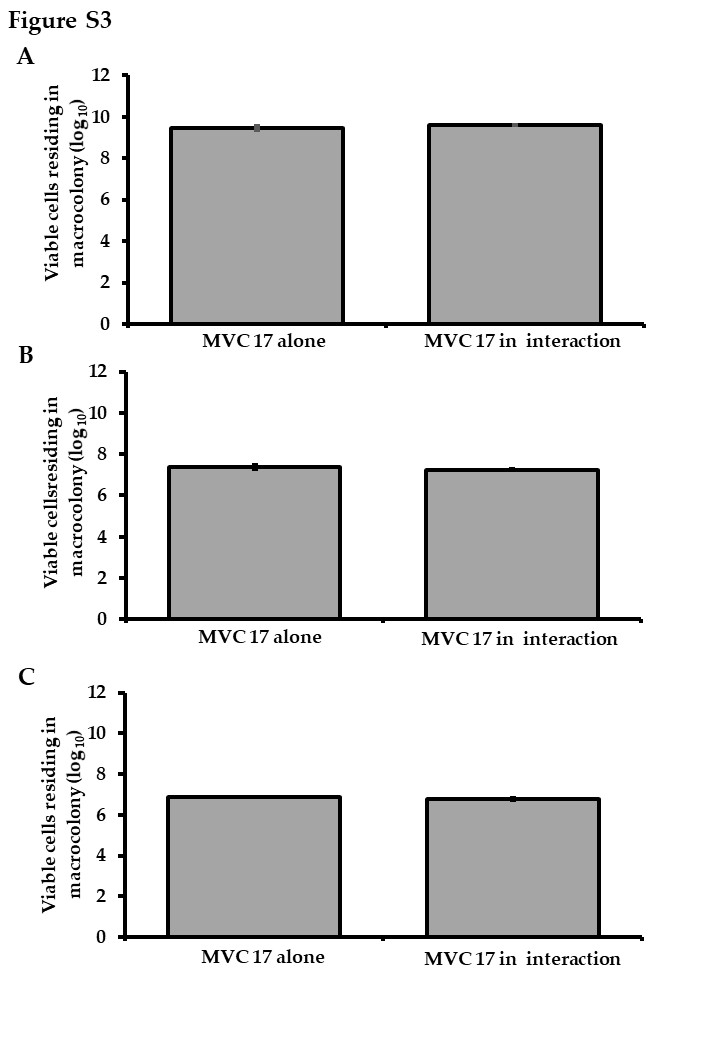

Supplement: Supplementary file 1 [file microorganisms-09-01186-s001.zip › Figure S3.jpg]

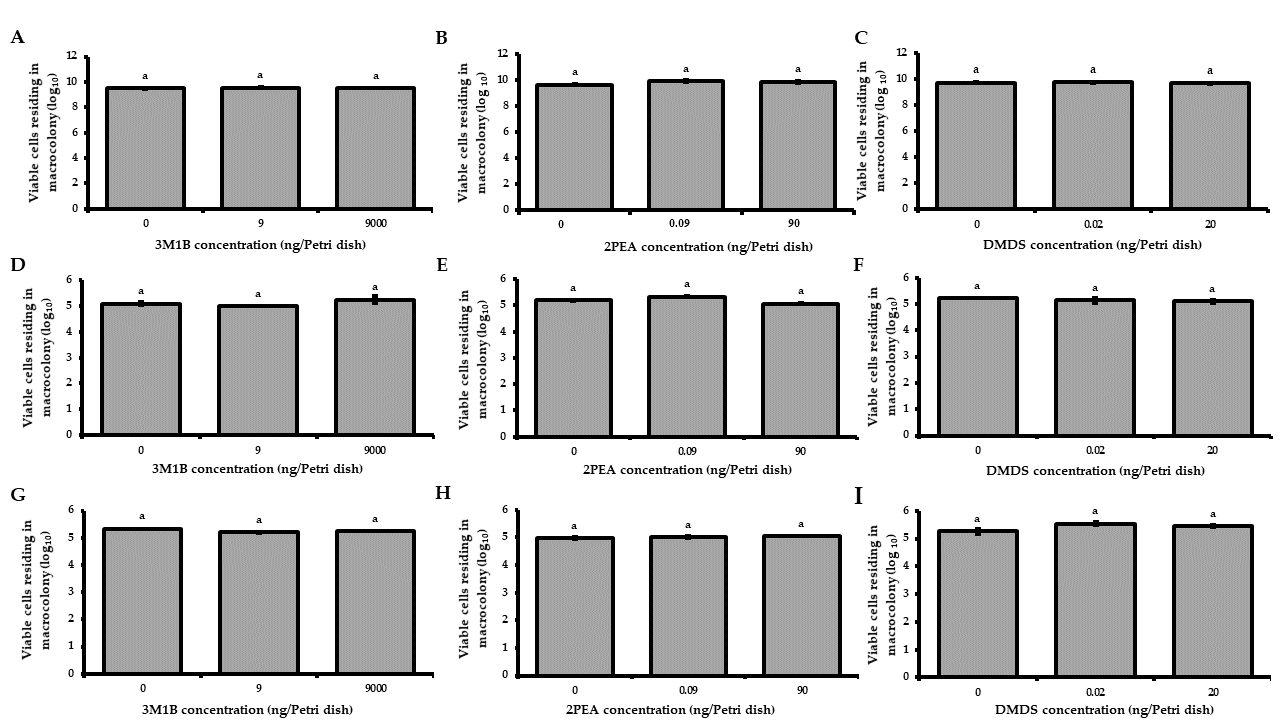

Supplement: Supplementary file 1 [file microorganisms-09-01186-s001.zip › Figure S4.gif]
